# Global niche dynamics of two invasive Aedes mosquitoes, Aedes japonicus and Aedes koreicus (Diptera: Culicidae), using comprehensive native and non-native occurrence data

**DOI:** 10.21203/rs.3.rs-9381631/v1

**Published:** 2026-05-21

**Authors:** Sangwoo Seok, Jiyeong Shin, Woo Jun Bang, Motoyoshi Mogi, Yoosook Lee

**Affiliations:** University of Florida; Seoul National University; Seoul National University; Saga University; University of Florida

**Keywords:** niche convergence, niche dynamics, species distribution models, invasive mosquitoes

## Abstract

**Background:**

Identifying areas at risk of invasion is essential for effective surveillance and management of invasive species. Two invasive mosquito species originating from East Asia, *Aedes japonicus* and *Aedes koreicus*, are of importance in Europe and America. Whether these invasive mosquitoes retain their environmental niches after introduction remains uncertain. Moreover, niche dynamics of these species, particularly for *Ae. koreicus*, remain poorly understood. This study aimed to quantify niche dynamics of both species by comparing environmental niches between native and non-native populations.

**Methods:**

We compiled occurrence records of *Ae. japonicus* and *Ae. koreicus* after from published sources, databases, and field collections, and applied data clearing and spatial filtering to reduce sampling bias. The final dataset included 2,623 for *Ae. japonicus* and 501 for *Ae. koreicus*. Environmental space was defined using selected bioclimatic variables and elevation. Within this space, we quantified niche overlap and dynamics using Schoener’s D and metrics of stability, expansion, and unfilling across species, regions, and invasion statues.

**Results:**

The two species occupied distinct niches in their native ranges but showed niche convergence in their non-native ranges. For both species, niche comparisons between native and non-native populations revealed subtle but significant niche differentiation. Niche dynamic analyses indicate high niche stability (0.821 in *Ae. japonicus* and 0.776 in *Ae. koreicus*) with low niche overlap values. Native subpopulation comparisons suggested potential invasion origins, with the Japanese population likely representing the source of *Ae. japonicus* and the Chinese population representing a potential additional source for *Ae. koreicus*.

**Conclusion:**

Our results show that these two invasive mosquito species exhibit convergent niche shifts after introduction. Niche comparisons between native and non-native populations show niche differentiation for both species and also provide insights into invasion origins. Incorporating comprehensive native occurrence data is critical for improving predictions of invasion risk and supporting surveillance and management of invasive mosquito species.

## Background

Identifying areas at risk of invasion is crucial for the effective surveillance and management of invasive species. Early detection and rapid response are key components of invasion management because interventions implemented at early stages of invasion are more likely to be successful and cost-effective [1]. For example, the early detection of *Aedes aegypti* in Australia was followed by rapid management actions, resulting in successful elimination [2]. Moreover, continuous surveillance of disease-associated mosquitoes is generally more cost-effective than responding after pathogen introduction, which often requires substantially higher management and response costs [3].

To aid early detection of invasive species in non-native ranges, species distribution models (SDMs) are widely used to predict invasion risk areas of invasive species [4–8]. These models typically combine occurrence data, particularly from the native range of the target species, with climate variables to estimate the realized environmental niche. Such projections rely on niche conservatism, which assumes that the target species maintain similar environmental niches when they establish in non-native ranges [9]. Therefore, before applying SDMs to predict their potential invasion areas, it is important to verify whether invasive species retain their environmental niche and whether the available data adequately represent their native distribution [10].

Two closely related *Aedes* mosquito species, *Aedes japonicus* (Theobald, 1901) and *Aedes koreicus* (Edwards, 1917), originate from East Asia [11, 12]. Both species are members of the Japonicus group, which also includes several species formerly regarded as subspecies of *Ae. japonicus* before being elevated to species status, such as *Ae. shintienensis* [13]. *Aedes japonicus* (often referred to as *Aedes japonicus japonicus* in earlier literature) has established invasive populations in America in 1998 and Europe in 2000 [11, 14]. *Aedes koreicus* has established invasive populations in Europe in 2008 [8, 15, 16]. Although neither species is currently regarded as a primary vector of arthropod-borne viruses (arboviruses) circulating in Europe and/or America, both *Ae. japonicus* and *Ae. koreicus* have demonstrated competence for several arboviruses in experimental studies, indicating their potential contribution to pathogen transmission [17, 18].

Although several SDM studies have examined the potential distribution of *Ae. japonicus* and *Ae. koreicus*, studies explicitly addressing their niche conservatism remain very limited [4–8]. One study has examined niche dynamics of *Ae. japonicus*, reporting high niche stability and low niche expansion during invasion [4]. However, the study did not define the native environmental niche using empirical occurrence data. Instead, the model assumed that *Ae. japonicus* is distributed across the entire Palearctic region of Japan and South Korea. This assumption may include environmentally unsuitable areas with extreme climatic, potentially leading to overestimation of the environmental niche occupied by the species in its native range. Consequently, the extent to which environmental niches differ between native and non-native populations of *Ae. japonicus* remains uncertain. In addition, to our knowledge, no study has yet investigated niche dynamics of *Ae. koreicus*. From a management perspective, such uncertainty can lead to inaccurate SDM-based risk assessment, thereby resulting in misidentification of areas at risk of invasion and inefficient surveillance and control strategies.

The inconsistency in niche dynamics reported in other invasive *Aedes* species makes it difficult to draw universal conclusions [19–21]. Some studies suggested a niche shift in *Ae. albopictus* [19, 21], whereas another study supported niche conservatism in *Ae. albopictus* [20]. One study suggested niche conservatism in *Ae. aegypti* [20], while a following study using a more comprehensive occurrence dataset again reported niche shifts in *Ae. aegypti* [21]. These contrasting findings suggest that conclusions about niche dynamics may be sensitive to the completeness and representativeness of occurrence data. Consequently, incorporating a comprehensive dataset is critical for accurately characterizing environmental niches and assessing niche dynamics during biological invasions.

To address this uncertainty, this study aims to compare environmental niches of native and non-native populations of *Ae. japonicus* and *Ae. koreicus* using comprehensive occurrence datasets. Specifically, we (1) evaluate niche differentiation between the two species, (2) examine geographic niche variation among native subpopulations, and (3) assess niche dynamics between native and non-native populations during invasions.

## Methods

### Occurrence records

Occurrence records of *Ae. japonicus* since 1980 were collected from research articles [22–113] and an online database [114]. Occurrence data for *Ae. koreicus* were obtained from datasets used in previous SDM studies [7, 8], as well as from subsequently published articles [30, 115, 116] and an online database [117]. In addition, we incorporated personal collection records of both species collected in Korea and Japan between 1980 and July 2025 by the authors (SS, JS, WJB, and MM). These specimens were obtained using various collection methods, including larval sampling, BG traps, aspiration, sweeping, and ovitraps. Sampling sites covered a wide range of environments, from urban areas to natural habitats, and from seacoasts to mountainous areas (up to 2,000 m).

We retained only coordinates reported to at least two decimal places in the WGS 84 geographic coordinate system, representing a spatial resolution on the order of hundreds of meters to about one kilometer. Duplicate records were subsequently removed. The compiled dataset included records of *Ae. japonicus* from the Oriental region, such as southern China, Hong Kong, Ryukyu Islands (Japan), and Philippines. However, *Ae. japonicus* is a species native to the Palearctic region [97], and records reported from the Oriental region are likely to represent other morphologically similar species belonging to the Japonicus group. Records of *Ae. japonicus* from southern China may reflect *Ae. shintienensis*, a species formerly treated as a subspecies of *Ae. japonicus* before being elevated to species status [13]. These records with taxonomic uncertainty were therefore excluded for both species. Several records of *Ae. koreicus* from Canada were removed because no published studies have documented the occurrence of *Ae. koreicus* in North America. To minimize potential sampling bias caused by high sampling density, we further applied spatial thinning by treating occurrence points located within 3 km of each other as duplicates, and only one record was retained using *spThin* package version 0.2.0 [118]. This threshold was chosen based on the average flight distance of female *Ae. japonicus* is approximately 2 km [119].

### Environmental space and principal components analysis

To define a unified geographical extent, we first combined occurrence coordinates of *Ae. japonicus* and *Ae. koreicus* from both native and non-native ranges. We then added a spatial buffer of 1 degree to the minimum and maximum values of longitude and latitude of the combined coordinates. The resulting extent defined the environmental extent for all subsequent analyses. Within the defined environmental extent, we randomly sampled 10,000 background points to represent background environmental conditions.

We obtained nineteen bioclimatic variables from the WorldClim database version 2.1 [120], representing average climatic conditions from 1970 to 2000. All climatic variables had a spatial resolution of 30 arc seconds (approximately 1 km at the equator). Elevation variable with 1 km resolution was obtained from the EarthEnv database [121], derived from the global digital elevation model GMTED2010 [122]. To reduce multicollinearity among the 20 variables, we calculated variance inflation factors (VIFs) using background environmental data and excluded variables with VIF values greater than 5. The remaining eight variables used for further analysis were Bio2, Bio3, Bio8, Bio9, Bio14, Bio15, Bio18, Bio19, Elevation (Supplementary Table S1).

We extracted the nine environmental variables at both background points and occurrence locations of the two species. Occurrence records lacking complete environmental data were excluded. Following the PCA-env framework [123], we performed principal component analysis (PCA) on the combined dataset of background and occurrence environmental variables to reduce dimensionality and define a shared environmental space. The first two principal components (PC1 and PC2) were retained because they explained the majority of environmental variance. We examined variable loadings on each principal component to interpret the environmental gradients represented by PC1 and PC2. This two-dimensional environmental space was subsequently used for all niche comparisons among species, populations, and subpopulations.

### Niche analyses

We evaluated niche relationships across multiple comparison types defined by species (*Ae. japonicus* and *Ae. koreicus*), distribution status (native and non-native), and geographic subdivision (Japan, Korea, China, America, and Europe). First, we conducted interspecific niche comparisons between *Ae. japonicus* and *Ae. koreicus* to assess overall niche differentiation between the two species. Second, we performed intraspecific comparisons between native subpopulations to evaluate geographic niche differentiation within each species. For *Ae. japonicus*, native populations were subdivided into Japanese and Korean subpopulations. For *Ae. koreicus*, native populations were subdivided into Chinese and Korean subpopulations. Third, we compared native and non-native populations within each species to assess niche conservatism during invasion. For *Ae. japonicus*, non-native populations were subdivided into American and European subpopulations, and each was compared with the native population. For *Ae. koreicus*, the non-native population was compared with the native population. Occurrence records from Russia Far East and North Korea were excluded from niche analysis because of insufficient sample size (N < 3).

**Figure 1:**
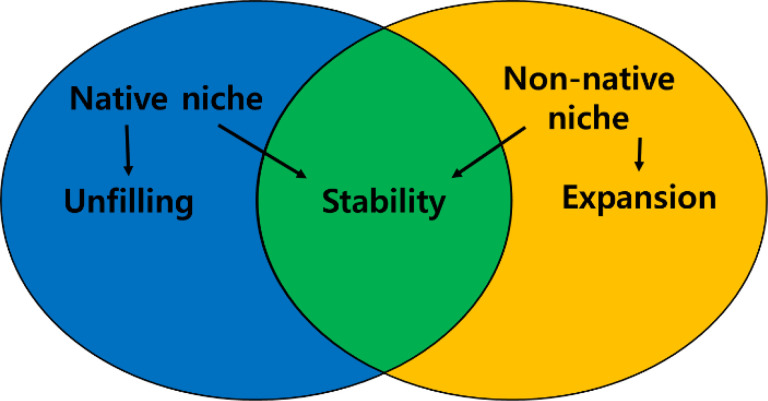
Conceptual illustration of three niche dynamics: unfilling, stability, and expansion.

Ecological niche overlaps were quantified using Schoener’s D, which measures the overlap between probability distribution of two niches in environmental space and ranges from 0 (no overlap) to 1 (complete overlap) [123]. Because niche overlap value alone does not fully describe how niches change during invasion, niche dynamics were additionally quantified using three complementary metrics: niche stability, niche expansion, and niche unfilling [124]. Niche stability represents the proportion of overlap between native and non-native niches relative to the non-native niche, niche expansion indicates the occupation of novel environmental conditions in the non-native ranges, and niche unfilling represents portions of the native niches that remain unoccupied in the non-native ranges relative to the native niche (Fig. 1).

We calculated niche overlap using Schoener’s D within a PCA-based environmental space. We also conducted niche equivalency tests and bidirectional niche similarity tests to determine whether observed niche overlaps differed from random expectations [125]. Niche dynamics (stability, expansion, and unfilling) were calculated for comparisons between native and non-native populations. All niche analyses were performed using *ecospat* package version 4.1.2 [126] in R version 4.5.0 [127].

## Results

### Occurrence records

**Figure 2:**
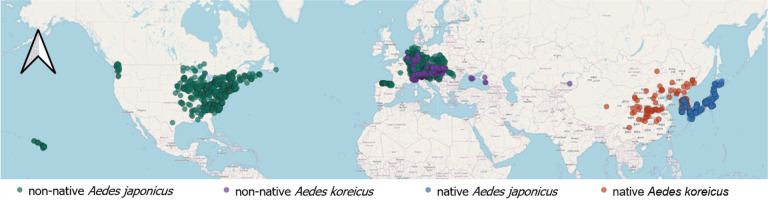
Global occurrence records of *Aedes japonicus* and *Aedes koreicus*. *Aedes japonicus* (native: blue, non-native: green) and *Aedes koreicus* (native: red, non-native: purple).

After all data preprocessing steps, the dataset comprised 2,623 occurrence records for *Ae. japonicus* (171 native and 2,452 non-native occurrence records) and 501 occurrence records for *Ae. koreicus* (138 native and 363 non-native occurrence records) (Supplementary Table S2) (Fig. 2). *Aedes japonicus* is distributed across its native range in Japan and Korean Peninsula and widely established throughout Europe and America. In this study, records from Hawaiian Islands were regarded as American population. *Aedes koreicus* is distributed across continental East Asia and parts of Europe. The distributions of the two species geographically overlap in the Korean Peninsula and in some parts of Europe.

### Environmental space and principal components analysis

**Figure 3:**
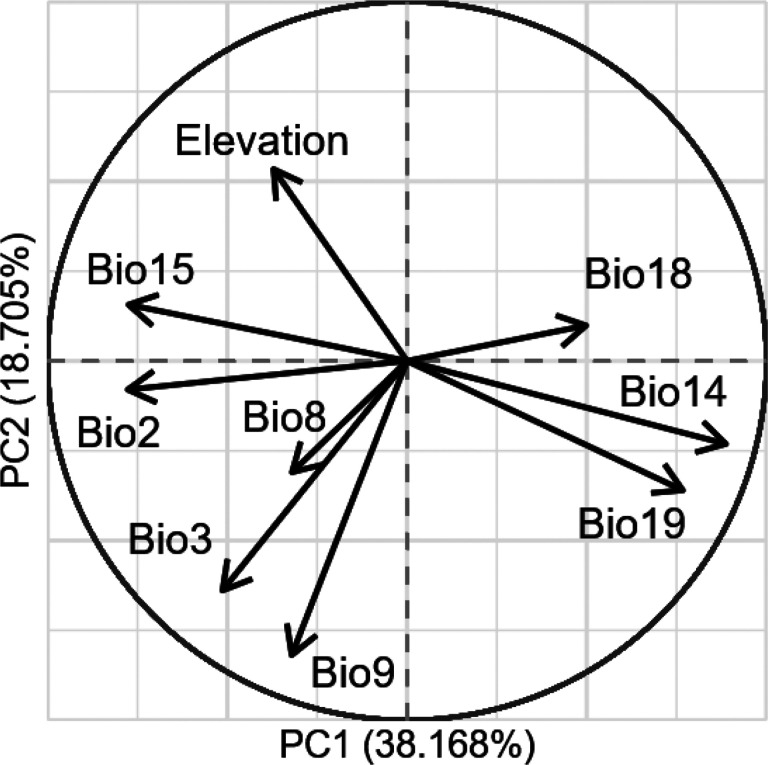
PCA correlation circle showing variable loadings on PC1 and PC2.

The two principal components captured 56.9% of environmental variance (PC1: 38.2%, and PC2: 18.7%) (Fig. 3). The three variables most strongly associated with PC1 were Bio14 (precipitation of driest month: 0.887), Bio2 (mean diurnal range: −0.777), and Bio15 (precipitation seasonality: −0.775). PC2 was mainly associated with Bio9 (mean temperature of driest quarter: −0.819), Bio3 (isothermality: −0.639), and Elevation (elevation: 0.534) (Supplementary Table S1). The environmental space defined by these two axes was used in subsequent niche comparisons between native and non-native populations of *Ae. japonicus* and *Ae. koreicus*.

#### Niche analysis between Ae. japonicus and Ae. koreicus

**Figure 4:**
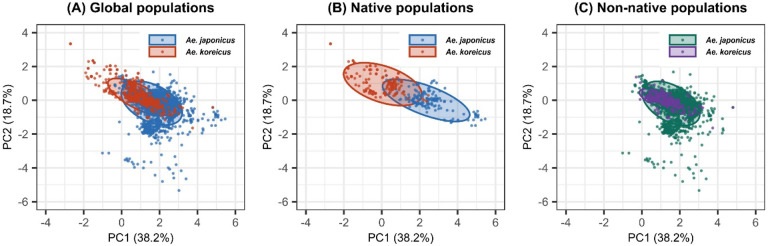
PCA-based environmental niche comparisons of *Aedes japonicus* and *Aedes koreicus*: (A) global populations; (B) native populations; (C) non-native populations. Shaded ellipses represent 95% confidence regions for each species.

Interspecific niche comparisons revealed contrasting patterns depending on whether native or non-native populations were included. When all global occurrence records were included, the two species showed high niche overlap (D = 0.424) with no evidence of niche differentiation, and similarity tests indicated significant niche similarity in both directions (p = 0.007 and p = 0.034) (Table 1). In native populations, significant niche differentiation was detected (D = 0.102, p < 0.001), whereas non-native populations showed no significant differentiation (D = 0.327, p = 0.138), with significant niche similarity in both directions (p = 0.016 and p = 0.035), indicating niche convergence in non-native ranges. Similar contrasting niche patterns were also observed in PCA-based environmental space. When non-native populations or all global populations were compared, the 95% confidence ellipses of the two species overlapped extensively (Fig. 4A, 4C). In contrast, when only native populations were considered, partial overlap was observed (Fig. 4B).

#### Niche analysis among Ae. japonicus subpopulations

**Figure 5:**
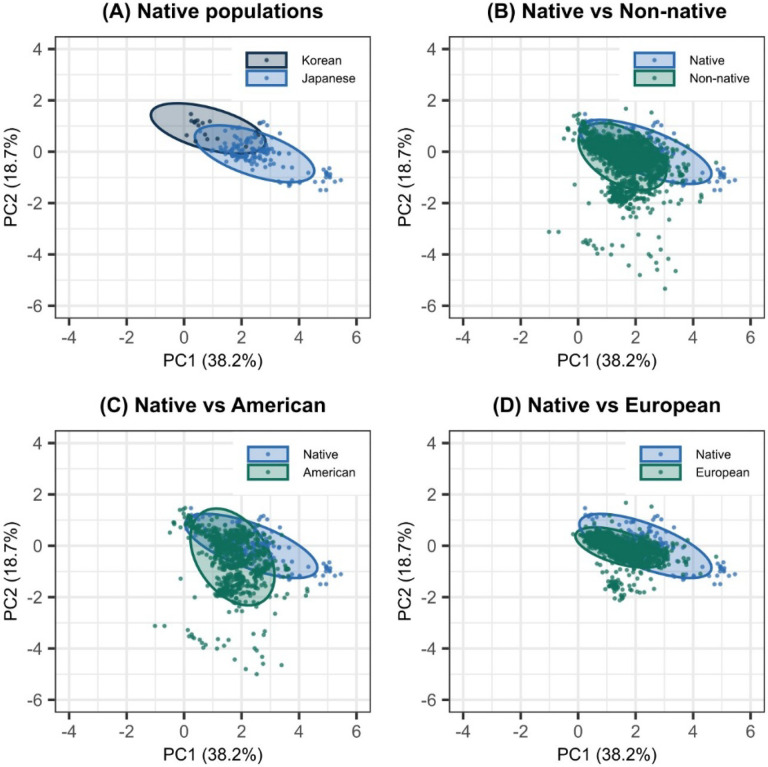
PCA-based environmental niche comparisons of *Aedes japonicus* population: (A) Japanese vs Korean populations; (B) native vs non-native populations; (C) native vs American populations; (D) native vs European populations; Shaded ellipses represent 95% confidence regions for each population.

Niche overlap analysis revealed clear differentiation among *Ae. japonicus* subpopulations. The Japanese and Korean native populations showed low niche overlap (D = 0.125), and the niche equivalency test indicated that their niches were significantly different (p < 0.001) (Table 2). Similarity tests were not significant in either direction (p > 0.05), suggesting that the niches of these native subpopulations were not more similar than from random distribution. Native subpopulations in PCA-based environmental space showed partial overlap, with the Japanese population extending toward positive values along PC1 and negative values along PC2 (Fig. 5A), indicating higher precipitation of driest month and coldest quarters.

Comparisons between native and non-native populations showed moderate niche overlap (D = 0.205–0.216), with equivalency tests consistently showing significant niche differentiation across all comparisons (p < 0.001) (Table 2). Niche dynamic analysis revealed high niche stability (0.693–0.909) and relatively low unfilling (0.095–0.192), indicating that a large portion of the native niche was retained in the non-native range. The 95% confidence ellipses of native and non-native populations of *Ae. japonicus* in PCA-based environmental space largely overlapped (Fig. 5B). In the non-native population, occurrences were more broadly distributed along the negative PC2 axis compared with the native populations. This pattern was more apparent when only the American *Ae. japonicus* population was considered (Fig. 5C), whereas the niche of the European *Ae. japonicus* population largely overlapped with the native *Ae. japonicus* population (Fig. 5D).

Comparisons among subpopulations generally showed low to moderate niche overlap (D = 0.106–0.226), and all equivalency tests indicated significant differences between niches of the subpopulations (Table 2). Notably, comparisons involving the Japanese *Ae. japonicus* population as a native subpopulation generally exhibited higher niche stability (0.711–0.829) than those involving the Korean *Ae. japonicus* subpopulation (0.221–0.326). Similarly, in PCA-based environmental space, the non-native *Ae. japonicus* population overlapped more with the Japanese *Ae. japonicus* population than with the Korean *Ae. japonicus* population (Fig. S1).

#### Niche analysis among Ae. koreicus subpopulations

**Figure 6:**
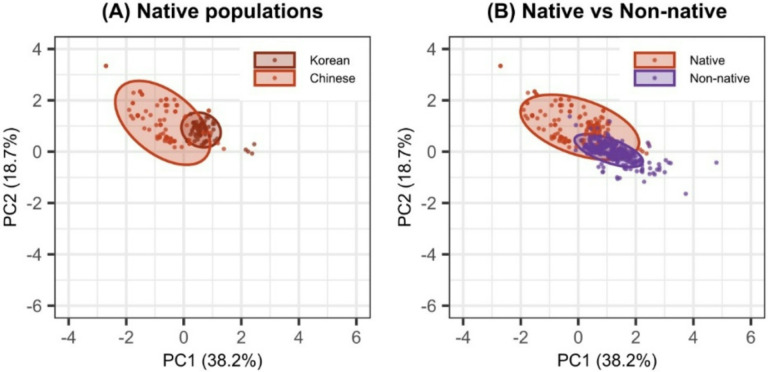
PCA-based environmental niche comparisons of *Aedes koreicus* population: (A) Chinese vs Korean populations; (B) native vs non-native populations. Shaded ellipses represent 95% confidence regions for each population.

Niche overlap analysis revealed clear differentiation between the native subpopulations of *Ae. koreicus*. The Chinese and Korean *Ae. koreicus* populations showed very low niche overlap (D = 0.066), and the niche equivalency test indicated that their niches were significantly different (p < 0.001) (Table 2). The similarity tests were not significant in either direction (p > 0.05), indicating that the niches of these native *Ae. koreicus* subpopulations were not more similar than expected. In PCA-based environmental space, the Korean *Ae. koreicus* population had a considerably narrower niche than the Chinese *Ae. koreicus* population (Fig. 6A).

Comparisons between native and non-native *Ae. koreicus* populations showed very low niche overlap (D = 0.083), and the equivalency test showed significant niche differentiation (p < 0.001) (Table 2). Niche dynamics analysis indicated moderate niche stability (0.776), with substantial niche unfilling (0.562) for *Ae. koreicus*. Most non-native *Ae. koreicus* occurrences fell within the native niche and were located relatively toward the positive side of PC1 and the negative side of PC2, suggesting that precipitation of driest month (Bio14) and precipitation of coldest quarter (Bio19) are most contributing variables for the non-native *Ae. koreicus* population (Fig. 6B).

Comparisons among *Ae. koreicus* subpopulations also showed low niche overlap (D = 0.049–0.144), and all equivalency tests indicated significant differences between niches (Table 2). Niche stability and expansion were similar between the Chinese and Korean *Ae. koreicus* subpopulations. Non-native *Ae. koreicus* occurrences did not fall clearly within either niche of native populations but instead overlapped to a similar extent with the niches of both the Chinese and Korean *Ae. koreicus* populations (Fig. S2).

## Discussion

### Environmental factors affecting distribution of Ae. japonicus and Ae. koreicus

Considering the variable loading and the distribution patterns in the PCA-based environmental space, niche differentiation between the two species is likely associated with elevation and precipitation-related variables: precipitation of driest month (Bio14), precipitation seasonality (Bio15), and precipitation of coldest quarter (Bio19) (Fig. 3). *Aedes japonicus* tended to occupy environments with higher precipitation during the driest month and coldest quarter and lower precipitation seasonality, indicating a preference for humid habitats with relatively stable moisture conditions throughout the year. In contrast, *Ae. koreicus* was more associated with higher seasonal precipitation and higher elevations. This pattern is well reflected in the distribution observed in Korea, where the two species co-occur but *Ae. koreicus* is predominant while *Ae. japonicus* is relatively rare [128]. The Korean Peninsula is characterized by its extensive mountainous terrain and highly seasonal precipitation, which closely align with the favorable environmental niche of *Ae. koreicus*.

### Niche convergence of Ae. japonicus and Ae. koreicus in their non-native ranges.

Although *Ae. japonicus* and *Ae. koreicus* occupy statistically different environmental niches within their native ranges, their niches partially overlap in environmental space (Fig. 4). This partial overlap indicates that the two species are not completely ecologically segregated in their native environments and may have facilitated the niche convergence of the two species observed after introduction into non-native habitat. When introduced into non-native regions, both species likely encountered climatic conditions that differ from those in their native habitats, potentially promoting rapid niche shifts [129]. In addition to these shifts, the observed convergence may also arise from shared ecological constraints among closely related species during establishment in non-native regions. Closely related taxa often exhibit similar physiological tolerances and climatic requirements due to phylogenetic niche conservatism [130]. Both *Ae. japonicus* and *Ae. koreicus* belong to the Japonicus Group [13] and share key ecological traits, including container-breeding life histories and association with temperate climates [11, 128]. Also, both species co-occur in the same habitats in parts of their native [128, 131] and non-native ranges [24]. These shared characteristics may restrict the range of environmental conditions that both species can successfully exploit, thereby producing overlapping realized niches in non-native regions. How stable their niches will be in non-native ranges and whether their range will shift closer to the native niche over a longer period of time in non-native ranges remains to be determined.

### Niche shift of Ae. japonicus and Ae. koreicus

Niche overlaps and dynamics reveal consistently high stability but low Schoener’s D across multiple population comparisons (Table 2). The high stability values indicate that invasive populations remain within the environmental niche space that their native populations occupy rather than expanding into non-native environmental ranges for *Ae. japonicus* and *Ae. koreicus*. Nonetheless, Schoener’s D values in this study, under previously proposed classification criteria [129], were classified as very limited overlap or low overlap, indicating subtle differences in environmental preferences between *Ae. japonicus* and *Ae. koreicus*. This implies that the specific combinations or gradients of environmental conditions preferred by non-native populations may have shifted. Such differentiation may reflect local adaptation during the establishment process in non-native regions [132]. Alternatively, non-native populations may originate from specific native subpopulations rather than representing the full native distributions, resulting in the occupation of only a subset of the native environmental niche [133].

In *Ae. japonicus*, niche dynamic analyses revealed slightly higher niche expansion in American populations than in European populations (Table 3), consistent with findings from previous niche study [4]. This pattern may be associated with multiple independent introduction events into Hawai i, eastern North America and western North America [37, 134], with distinct genetic lineages potentially contributing to a broader environmental niche range.

In *Ae. koreicus*, the non-native population in Europe occupied a much narrower niche range than the native population, with low expansion and high unfilling values (Table 3). This pattern implies that only a small portion of the native population was introduced, resulting in occupation of only a limited part of the entire native niche. Alternatively, the shorter invasion history of *Ae. koreicus* (first report in 2008) compared to *Ae. japonicus* (first report in 1998) may explain high unfilling [14, 15]. Of note, this species has not been observed in the American continent as of March 2026. The high unfilling value may indicate the potential for further expansion as invasion progresses [125].

### Inference on source population of Ae. japonicus and Ae. koreicus

Niche analysis can provide insights into the identification of potential source populations [135]. For *Ae. japonicus*, higher stability values of the Japanese subpopulation compared to the Korean subpopulations may suggest that the Japanese *Ae. japonicus* subpopulation represent a potential source of the non-native *Ae. japonicus* subpopulations in America and Europe (Table 2). This is consistent with previous findings that North American populations of *Ae. japonicus* are genetically more closely related to the Japanese population than to the Korean population [136].

For *Ae. koreicus*, our results raise questions about the prevailing hypothesis that the European population originated solely from the two Korean *Ae. koreicus* populations: the Korean mainland and Jeju Island [15, 16]. In this study, the Chinese *Ae. koreicus* population exhibited slightly higher stability values than the Korean population, suggesting that it may represent an additional potential source of European populations (Table 2).

To date, genetic reference data representing native populations have been largely limited to a small number of Korean samples of two species [136, 137], and these datasets are predominantly based on analyses of a restricted set of genetic markers. The limited representation of native populations, together with the inherent limitations of marker-based approaches, suggests that previous interpretations regarding the origin of European invasive *Ae. koreicus* populations may have been influenced by incomplete geographic sampling Future studies incorporating whole-genome comparisons across multiple native populations will therefore be essential for accurate inference of the origins of invasive *Ae. koreicus* populations.

#### Influence of underlaying data on the niche prediction

Our results underscore the importance of incorporating native occurrence data for accurate prediction of the potential global distribution of these two invasive species. Expansion values of 0.179–0.224 observed in comparisons between native and non-native populations indicate that having non-native occurrence records alone do not fully capture the environmental niche of the native range (Table 2). Despite this importance, previous studies have been limited by the unavailability of reliable native occurrence data. Native occurrence records for *Ae. japonicus* were overrepresented in native ranges [4], and those for *Ae. koreicus* have been constrained by a limited number of occurrence records given their wide geographic extent [7, 8]. To address these limitations, we compiled a comprehensive dataset of native occurrences by integrating published literature, public databases, and previously unpublished field collections conducted by the authors across Korea and Japan. In particular, we incorporated records from literature that have been underutilized in previous studies due to language barriers. At the same time, we carefully evaluated the taxonomic validity of the records to ensure that the occurrence data for both species were compiled as accurately as possible. By incorporating native occurrence data that were previously underutilized, our study provides a more comprehensive characterization of the environmental niche of these two important invasive mosquito species that have crossed continental boundaries. These improved niche representations can enhance not only SDM performance, but also other niche-based predictive modelling frameworks used to support surveillance and management of invasive mosquito species.

Despite our effort, this study may not have fully captured the full extent of native niche. Occurrence records from North Korea are extremely limited. However, given that both species are present in South Korea, it is highly likely that the two species also occur in North Korea. Therefore, this limitation may lead to incomplete representation of the native niche. In addition, the distribution of *Ae. japonicus* in China and Russia remains uncertain. Literature from the 1980s and 1990s reported its presence in these regions [138–140], but it is unclear whether these records represent *Ae. japonicus* or other species of the Japonicus group that were previously treated as subspecies. As we couldn’t find subsequent records confirming the presence of *Ae. japonicus* in China and Russia, we assumed that these earlier records likely represent other species within the Japonicus group, such as *Ae. shintienensis*. Clarifying the distributional status of species of the Japonicus group would improve understanding of niche dynamics and prediction.

## Conclusion

This study examined niche shift and dynamics in two closely related invasive mosquito species, *Ae. japonicus* and *Ae. koreicus*, which represent rare cases of mosquitoes crossing continental boundaries and establishing in non-native regions. Our results revealed three main findings. First, although the two species occupied distinct environmental niches in their native ranges, their niches showed convergence in the non-native ranges. Second, non-native populations of both species occupied niches that differed significantly from those of their native populations. Subtle but significant differences in environmental preferences between native and non-native populations imply that invasive populations may represent only a subset of the native niche or may undergo local adaptation following introduction. Third, specific native subpopulations may have contributed to shaping the niches of the non-native populations. Higher niche stability between Japanese and non-native populations of *Ae. japonicus* supports Japan as a source region. In contrast, results for *Ae. koreicus* suggest that Chinese populations may represent an additional potential source for the European populations. Overall, incorporating comprehensive native occurrence data is essential for accurately characterizing environmental niches and improving predictive models used for surveillance and management of invasive mosquito species.

## Supplementary Material

Supplementary Files

This is a list of supplementary files associated with this preprint. Click to download.

- TableS1.xlsx
- TableS2.xlsx
- FigureS2.svg
- FigureS1.svg

## Figures and Tables

**Table 1:** Interspecific niche overlaps and statistical tests between *Aedes japonicus* and *Aedes koreicus* across global, native, and non-native populations. Schoener’s D (D) indicates niche overlap (0 = no overlap, 1 = complete overlap). Equivalency (Eq.) tests assess whether the niches of the two species are statistically equivalent. Similarity (Sim.) tests evaluate whether niche overlap is greater than expected by chance in both directions: from *Ae. japonicus* to *Ae. koreicus* (1→2) and from *Ae. koreicus* to *Ae. japonicus* (2→1).

*Aedes japonicus*	*Aedes koreicus*	D	Eq. p value	Sim. (1→2) p value	Sim. (2→1) p value
Global	Global	0.424	1	0.007	0.034
Native	Native	0.102	<0.001	0.237	0.456
Non-native	Non-native	0.327	0.1379	0.016	0.035

**Table 2:** Intraspecific niche overlaps and statistical tests between subpopulations of *Aedes japonicus* and *Aedes koreicus*. Schoener’s D (D) indicates niche overlap (0 = no overlap, 1 = complete overlap). Equivalency (Eq.) tests assess whether the niches of the two species are statistically equivalent. Similarity (Sim.) tests evaluate whether niche overlap is greater than expected by chance in both directions: from the first population (Pop 1) to the second population (Pop 2) (1→2) and from Pop 2 to Pop 1 (2→1).

Pop 1	Pop 2	D	Eq. p value	Sim. (1→2) p value	Sim. (2→1) p value	Stability	Expansion	Unfilling
*Aedes japonicus*								
Japanese	Korean	0.125	<0.001	0.914	0.388			
Native	Non-native	0.205	<0.001	0.541	0.747	0.821	0.179	0.099
Native	American	0.215	<0.001	0.402	0.613	0.693	0.307	0.095
Native	European	0.216	<0.001	0.684	0.837	0.909	0.091	0.192
Japanese	Non-native	0.209	<0.001	0.544	0.729	0.829	0.171	00.107
Japanese	American	0.186	<0.001	0.465	0.686	0.711	0.289	0.115
Japanese	European	0.226	<0.001	0.682	0.768	0.916	0.084	0.178
Korean	Non-native	0.122	0.003	0.392	0.882	0.278	0.722	0.035
Korean	American	0.134	0.003	0.294	0.691	0.221	0.779	0.012
Korean	European	0.106	<0.001	0.208	0.985	0.326	0.674	0.298
*Aedes koreicus*								
Chinese	Korean	0.066	<0.001	0.736	0.647			
Native	Non-native	0.083	<0.001	0.511	0.759	0.776	0.224	0.562
Chinese	Non-native	0.049	<0.001	0.406	0.878	0.522	0.478	0.827
Korean	Non-native	0.144	<0.001	0.381	0.881	0.456	0.544	0.195

## Data Availability

The occurrence data used in this study are available in the Supplementary Table.
